# Autoimmune rheumatic diseases in women with coronary microvascular dysfunction: a report from the Women's Ischemia Syndrome Evaluation—Coronary Vascular Dysfunction (WISE-CVD) project

**DOI:** 10.3389/fcvm.2023.1155914

**Published:** 2023-05-30

**Authors:** Melanie T. Chen, Joseph Chang, Ashley S. Manchanda, Galen Cook-Wiens, Chrisandra L. Shufelt, R. David Anderson, John W. Petersen, Dhaval R. Naik, Louise E. J. Thomson, Daniel S. Berman, Eileen M. Handberg, Carl J. Pepine, C. Noel Bairey Merz, Janet Wei

**Affiliations:** ^1^ Barbra Streisand Women's Heart Center, Cedars-Sinai Smidt Heart Institute, Los Angeles, CA, United States; ^2^ Biostatistics Research Center, Cedars-Sinai Medical Center, Los Angeles, CA, United States; ^3^ Division of Internal Medicine, Women's Health Research Center, Mayo Clinic, Jacksonville, FL, United States; ^4^ Division of Cardiovascular Medicine, University of Florida College of Medicine, Gainesville, FL, United States; ^5^ Mark S. Taper Imaging Center, Cedars-Sinai Medical Center, Los Angeles, CA, United States

**Keywords:** autoimmune rheumatic diseases, coronary microvascular dysfunction, coronary vasospasm, chest pain, ischemic heart disease

## Abstract

**Background:**

While autoimmune rheumatic diseases (ARDs) have been linked with coronary microvascular dysfunction (CMD), the relationship between ARD and CMD in women with signs and symptoms of ischemia and no obstructive arteries (INOCA) are not well described. We hypothesized that among women with CMD, those with ARD history have greater angina, functional limitations, and myocardial perfusion compromise compared to those without ARD history.

**Methods:**

Women with INOCA and confirmed CMD by invasive coronary function testing were included from the Women's Ischemia Syndrome Evaluation-Coronary Vascular Dysfunction (WISE-CVD) project (NCT00832702). Seattle Angina Questionnaire (SAQ), Duke Activity Status Index (DASI), and cardiac magnetic resonance myocardial perfusion reserve index (MPRI) were collected at baseline. Chart review was performed to confirm self-reported ARD diagnosis.

**Results:**

Of the 207 women with CMD, 19 (9%) had a confirmed history of ARD. Compared to those without ARD, women with ARD were younger (*p* = 0.04). In addition, they had lower DASI-estimated metabolic equivalents (*p* = 0.03) and lower MPRI (*p* = 0.008) but similar SAQ scores. There was a trend towards increased nocturnal angina and stress-induced angina in those with ARD (*p* = 0.05 for both). Invasive coronary function variables were not significantly different between groups.

**Conclusions:**

Among women with CMD, women with a history of ARD had lower functional status and worse myocardial perfusion reserve compared to women without ARD. Angina-related health status and invasive coronary function were not significantly different between groups. Further studies are warranted to understand mechanisms contributing to CMD among women with ARDs with INOCA.

## Introduction

Autoimmune rheumatic diseases (ARDs), including systemic lupus erythematosus (SLE), rheumatoid arthritis (RA), systemic vasculitis, spondyloarthropathy (e.g., ankylosing spondylitis and psoriatic arthritis), and systemic sclerosis, have been associated with increased cardiovascular morbidity and mortality (1). Previous studies have focused on the role of systemic inflammation in these diseases promoting accelerated and premature atherosclerosis leading to macrovascular coronary artery disease (CAD) (2). Systemic inflammation also promotes endothelial dysfunction leading to coronary microvascular dysfunction (CMD) and is a distinct pathway for macrovascular and microvascular cardiac involvement in patients with ARDs (3–5). CMD is frequently present in patients with signs and symptoms of ischemia and no obstructive arteries (INOCA) (6). The prevalence of ARDs in women with signs and symptoms of INOCA, and relationship of ARDs to CMD and CMD-related outcomes, however, have not been well described.

The Women's Ischemia Syndrome Evaluation—Coronary Vascular Dysfunction (WISE-CVD) Project has previously shown that evidence of CMD predicts major adverse cardiovascular outcomes (7, 8). We hypothesized that among women with CMD, those with ARD history would have greater comorbidities, cardiac functional and structural abnormalities, and worse CMD-related outcomes including greater angina burden and limited functional status, than those without ARD history.

## Methods

Women were enrolled in the National Heart, Lung, and Blood Institute–sponsored WISE-CVD study (NCT00832702) after invasive coronary angiography performed to further evaluate symptom and/or signs of ischemia documented no obstructive CAD (defined as <50% diameter stenosis in epicardial arteries) as previously described (9).

All women underwent baseline evaluation, including standardized collection of demographic variables, risk factors, medical history, medication use, symptom history, physical examination, labs, Seattle Angina Questionnaire (SAQ) (10, 11) and Duke Activity Status Index (DASI) (12). Women with self-reported autoimmune disease history available were identified by review of medical records to confirm history of ARD and whether patients were treated with systemic or biologic agents. A history of ARD was defined as having any of the following: rheumatoid arthritis (RA), spondyloarthropathies (such as ankylosing spondylitis and psoriatic arthritis), systemic lupus erythematosus (SLE), systemic vasculitides, inflammatory myopathies, mixed connective tissue disease, systemic sclerosis, or Sjogren's syndrome.

Coronary function testing was performed in a clinically indicated subset, per previously published protocol (9, 13). Coronary flow reserve (CFR) was calculated as a ratio of average peak velocity to average baseline velocity in response to adenosine. Coronary blood flow change (*Δ*CBF) and change in coronary artery diameter (*Δ*ACH) were measured in response to acetylcholine. Coronary diameter response was measured in response to nitroglycerin (NTG) (9). Coronary function variables were interpreted by the WISE Coronary Core Laboratory, masked to clinical data (13). CMD was defined as a CFR <2.5, *Δ*CBF <50%, *Δ*ACH ≤0%, or *Δ*NTG <20% (9), representing non-endothelial microvascular dysfunction, endothelial microvascular dysfunction, non-endothelial macrovascular dysfunction, and endothelial macrovascular dysfunction, respectively. The WISE coronary artery disease severity score, a measure of coronary atherosclerotic burden, was measured per protocol (14).

Cardiac magnetic resonance (CMR) imaging was conducted according to a standardized protocol used for assessment of left ventricular (LV) morphology and function, pharmacologic stress first-pass myocardial perfusion imaging, and delayed contrast enhancement, as previously described (9). Pharmacologic stress was performed with adenosine or regadenoson. First-pass perfusion images were obtained in basal, mid, and distal short-axis image planes. The WISE CMR core lab analyzed myocardial perfusion reserve index (MPRI), LV mass, LV volumes, LV early peak filling rate, and time-to-peak filling rate by manually tracing the epicardial and endocardial borders of the short-axis cine images and indexed to body surface area, as previously described (9). The stress vs. rest upslope was normalized to LV cavity input for the calculation of MPRI.

**Figure 1:**
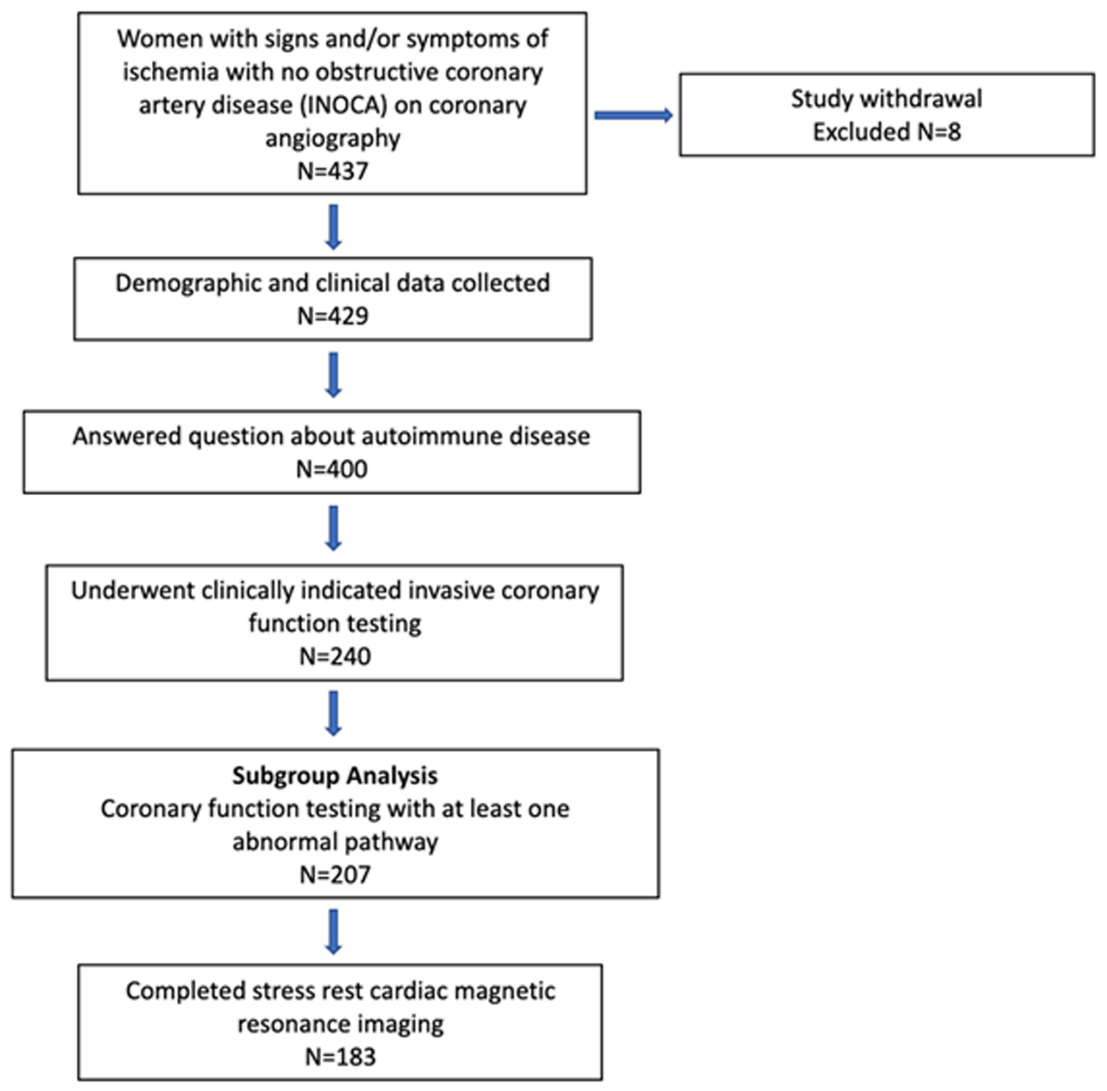
WISE-CVD ARD study schematic. Study design with actual number of women enrolled and included in each subgroup.

**Figure 2:**
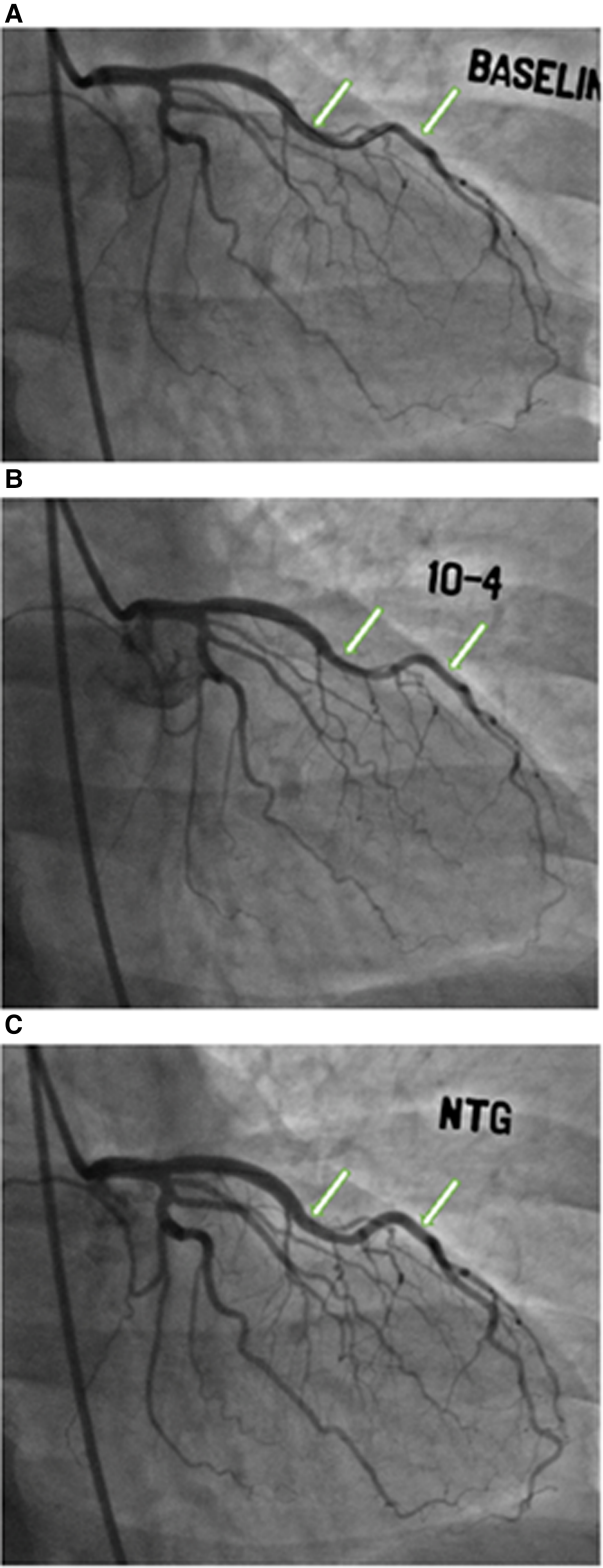
Coronary function testing of a participant with ankylosing spondylitis. Arrows show abnormal vasoconstriction of left anterior descending artery after acetylcholine administration (**B**) compared to baseline (**A**), with vasodilation after nitroglycerin administration (**C**)

The study design is summarized in Figure 1. Of the 437 women enrolled in the WISE-CVD cohort, 400 answered the ARD question, 240 underwent clinically indicated invasive coronary function testing, and 207 had at least one abnormal invasive coronary function variable indicating CMD. Of the 207 patients with CMD detected on invasive coronary function testing, 19 (9%) had a confirmed history of ARD, and 183/207 (88%) completed the baseline CMR. Figure 2 illustrates a representative abnormal coronary function testing result of a participant with ARD.

Variables were summarized using counts and percentages for categorical variables and means and standard deviations for quantitative variables. ARD status from chart review determined the two groups. A difference in average age was found using a two-group *t*-test. Age-adjusted tests between the groups came from logistic regression models with autoimmune disease status as the outcome and age and the row variable as explanatory variables. The age-adjusted *p*-values were the regression coefficient chi-squared tests or type 3 chi-squared tests of the multi-category variables in the models. A significance level of *p* < 0.05 was used for all tests.

## Results

Table 1 summarizes pertinent demographics and baseline clinical characteristics of the 207 women with confirmed CMD. Of the 207 women with confirmed CMD, 19 had confirmed ARD (9%), with the most prevalent diagnoses being SLE (*n* = 13, 68%) and RA (*n* = 3, 16%). Of the 15/19 ARD patients who reported taking corticosteroids and disease-modifying antirheumatic drugs, treatment regimens included corticosteroids (*n* = 6), hydroxychloroquine (*n* = 8), methotrexate (1), rituximab (1), mycophenolate (1), leflunomide (1) and cyclophosphamide (1). Compared to women without ARD, women with ARD were younger and had lower fasting glucose levels, but otherwise there were no significantly different baseline characteristics. Women with ARD were found to have similar angina-related health status, as measured by SAQ, compared to women without ARD. However, those with ARD reported a trend towards increased nocturnal angina and increased angina with exertion or emotional stress. Lastly, women with ARD had lower functional capacity as estimated by DASI.

**Table 1:** Baseline clinical characteristics.

Variable	No History of ARD (*N* = 188)	History of ARD (*N* = 19)	Total (*N* = 207)	Age-Adjusted *P*-value
Age, years	54.4 ± 11.5	48.9 ± 8	53.9 ± 11.4	–
BMI, kg/m^2^	28.6 ± 7.5	28.4 ± 6.3	28.6 ± 7.4	0.96
Caucasian	139 (73.9%)	13 (68.4%)	152 (73.4%)	0.83
Hypertension	69 (39.2%)	8 (42.1%)	95 (48.5%)	0.33
Dyslipidemia	29 (20.4%)	2 (16.7%)	31 (20.1%)	0.90
Diabetes	22 (12.0%)	1 (5.3%)	23 (11.3%)	0.44
Any Smoking	84 (44.7%)	5 (26.3%)	89 (43.0%)	0.12
Renal Disease	2 (1.1%)	0 (0.0%)	2 (1.0%)	0.99
Depression	44 (23.9%)	2 (10.5%)	46 (22.7%)	0.19
Migraines	91 (48.4%)	11 (57.9%)	102 (49.3%)	0.61
DASI-estimated METs	8.0 ± 5.7	5.3 ± 4.1	7.8 ± 5.6	**0**.**03**
SAQ-7 Summary Score	58.1 ± 21.8	57.6 ± 22.4	58.0 ± 21.8	0.82
SAQ Physical Limitation Score	65.3 ± 24.6	66.0 ± 23.6	65.4 ± 24.5	0.91
SAQ Angina Frequency Score	62.6 ± 26.0	57.4 ± 28.8	62.1 ± 26.2	0.60
SAQ Angina Stability Score	45.7 ± 27.6	38.2 ± 26.8	45.0 ± 27.5	0.23
SAQ Treatment Satisfaction Score	70.2 ± 23.5	79.5 ± 21.0	71.1 ± 23.4	0.07
SAQ Quality of Life Score	47.4 ± 23.9	47.4 ± 22.4	47.4 ± 23.7	0.63
Angina with Nighttime Awakenings	62 (35.8%)	12 (63.2%)	74 (38.5%)	0.05
Stress-Induced Chest Pain	103 (57.5%)	15 (78.9%)	118 (59.6%)	0.05

Data in *n* (%) or mean ± SD.

ARD, autoimmune rheumatic disease; BMI, body mass index; *Δ*ACH, change in coronary artery diameter to acetylcholine; *Δ*CBF, change in coronary blood flow to acetylcholine; CFR, coronary flow reserve; DASI, Duke Activity Status Index; METs, metabolic equivalents; MPRI, myocardial perfusion reserve index; *Δ*NTG, change in coronary artery diameter to nitroglycerin; SAQ-7, Seattle Angina Questionnaire (7-item), scores range from 0 to 100 with higher scores indicating better angina status.

CMR and invasive coronary function testing measurements are shown in Table 2, stratified by ARD history. MPRI was lower in ARD patients compared to non-ARD patients, while there was no difference in LV structure, systolic or diastolic function, or prevalence of late gadolinium enhancement. Invasive coronary function testing variables did not differ significantly between those with and without a history of ARD. There were similar coronary atherosclerosis severity scores between the two groups. There was a trend towards a lower CFR and increased prevalence of CFR <2.5 in ARD patients. All other measures of coronary function, including coronary blood flow change, diameter response to acetylcholine, nitroglycerin response, provoked coronary artery vasospasm and left ventricular end-diastolic pressure also did not differ significantly between ARD and non-ARD patients.

**Table 2:** Invasive coronary function testing and cardiac magnetic resonance imaging.

Invasive Coronary Function Testing Results			
Measurement	No History of ARD (*N* = 188)	History of ARD (*N* = 19)	Age Adjusted *P*-value
Coronary Severity Score	9.6 ± 4.2	9.5 ± 4.3	0.87
LV End-Diastolic Pressure at rest, mmHg	14.4 ± 5.2	13.3 ± 4.3	0.51
CFR	2.7 ± 0.7	2.4 ± 0.5	0.07
CFR < 2.5	77 (43.5%)	11 (68.8%)	0.08
*Δ*CBF, %	53.9 ± 74.9	58.2 ± 72.1	0.93
*Δ*CBF < 50%	93 (61.6%)	9 (52.9%)	0.60
*Δ*ACH, %	−3.0 ± 14.3	−0.6 ± 13.6	0.63
*Δ*ACH ≤ 0%	96 (55.2%)	11 (64.7%)	0.36
*Δ*NTG, %	12.2 ± 12.4	11.5 ± 11.5	0.48
*Δ*NTG < 20%	130 (75.1%)	14 (77.8%)	0.54
Coronary Artery Vasospasm	12 (6.4%)	1 (5.3%)	0.96
Cardiac Magnetic Resonance Imaging Results			
Measurement	No History of ARD (*N* = 166)	History of ARD (*N* = 17)	Age Adjusted *P*-value
LV Ejection Fraction, %	67.7 ± 7.0	68.8 ± 8.3	0.28
LV End-Diastolic Volume Index, ml/m^2^	69.9 ± 11.3	68.2 ± 12.4	0.20
LV End-Systolic Volume Index, ml/m^2^	22.8 ± 6.9	21.3 ± 6.5	0.11
LV Mass Index, ml/m^2^	52.0 ± 7.3	51.8 ± 5.8	0.89
Presence of late gadolinium enhancement	8 (5.0%)	1 (6.2%)	0.86
Peak Filling Rate, ml/s	366.4 ± 102.0	376 ± 101.9	0.67
Time-to-Peak Filling Rate, ms	195.6 ± 56.6	180.6 ± 36.3	0.47
MPRI	1.9 ± 0.5	1.6 ± 0.5	**0**.**008**

Data in *n* (%) or mean ± SD.

ARD, autoimmune rheumatic disease; CFR, coronary flow reserve in response to adenosine; *Δ*CBF, change in coronary blood flow in response to acetylcholine; *Δ*ACH, change in coronary artery diameter in response to acetylcholine; *Δ*NTG, change in coronary artery diameter in response to nitroglycerin; LV, left ventricular;

MPRI, myocardial perfusion reserve index.

## Discussion

Our study supports that a history of ARD among women with INOCA and confirmed CMD have worse functional status, reduced myocardial perfusion reserve, and a trend towards more angina, however angina-related health status and invasive coronary function were not significantly different compared to women without ARD. To the best of our knowledge, this is the first study to report invasive coronary function testing in women with INOCA and confirmed CMD with ARD.

Patients with ARD are known to be at increased risk of cardiovascular disease compared to the general population (4). Their risk cannot be completely explained by traditional risk factors, such as hyperlipidemia, obesity, and hypertension but has been suggested to be related to the duration of the ARD, as well as overlap among the ARDs. For example, patients with SLE and no history of CAD were found to have a ten times higher risk of cardiovascular disease compared to predictions based on traditional risk factors alone (15). In the absence of obstructive CAD, ARD patients may have increased risk for major adverse cardiac events associated with CMD (16). A previous study found that when compared to reference subjects, patients with SLE and chest pain were more likely to have abnormal CMR stress perfusion and reduced MPRI consistent with CMD (17). Similarly, patients with systemic sclerosis, RA, or psoriasis have worse microvascular function, as measured by noninvasive CFR, when compared to healthy controls (18–20). Since traditional stress testing has poor sensitivity for diagnosing CMD (21, 22), the prevalence of CMD in ARD populations is not well understood.

Mechanisms underlying CMD may be due to inflammation, as severity of microvascular impairment has been shown to correlate with severity and duration of RA, systemic sclerosis, and SLE, even in asymptomatic patients without cardiovascular risk factors (18, 19). Moreover, prospective cohort studies have shown that patients treated with biologic therapy have significant reduction in coronary inflammation and total plaque burden, which can impact coronary vasomotor function (23, 24). The pro-inflammatory state in ARD can inhibit normal coronary microvascular function and myocardial blood flow regulatory mechanisms, contributing to an increased risk for myocardial ischemia and long-term cardiovascular events (3). Our results are consistent with a previous investigation of women with INOCA demonstrating that non-endothelial function, as measured by noninvasive myocardial blood flow reserve, was negatively associated with four inflammatory biomarkers (25).

Our findings showed significantly lower noninvasive CMR MPRI in the ARD group, however no invasive coronary function variables were significantly different. A previous investigation has shown modestly significant positive correlations between individual coronary function variables and MPRI (26). CMD is a heterogenous disorder of the coronary microvasculature perhaps best measured by global MPRI compared to invasive testing of a single coronary artery as conducted. Further, CMD encompasses a variety of coronary endothelial and non-endothelial reactivity dysfunction. It is possible that the CMD seen in ARD is mediated by different pathways with no particular pathway playing a predominant role. For example, it has been proven that at least three separate pathways, mediated by increased oxidative stress, pro-inflammatory CD4 ^+ ^CD28^null^ T-cells, and monocyte dysregulation each play a role in ARD-mediated atherosclerosis and coronary microvascular disease (3). Therefore, patients with ARD may have significantly worse myocardial perfusion reserve without any single pathway achieving a statistically significant level of difference, compared to patients without ARD.

Despite worse myocardial perfusion reserve, women with CMD and with ARD vs. no ARD had similar angina-related health status. A previous cohort study of patients undergoing serial ischemia assessments has shown no correlation between the changes in the magnitude of ischemia measured by stress echocardiography, which is insensitive to CMD (21) and SAQ angina frequency (27). Our results demonstrated a trend towards a relatively higher burden of stress-induced chest pain in patients with ARD, which may be cardiac, non-cardiac, or a spurious finding. Interestingly, cross-sectional studies have shown elevated work-related stress in patients with SLE and RA compared to matched controls (28). Mental-stress associated ischemia has been shown to be mediated by CMD (29) and may not be reflected in pharmacologic stress testing. Repeated mental stress ischemic episodes and elevation in pulsatile LV loading may ultimately impair LV function and reduce diastolic pressure time fraction and subsequently contribute to decreased myocardial perfusion (30). The specific inflammatory pathways that predispose ARD patients to cardiovascular disease at an accelerated rate may also increase their rate of stress-related angina.

The ARD women had significantly worse functional capacity compared to those without ARD, although this did not appear to be related to angina given no difference in the SAQ physical limitation scale. Previous studies have shown that INOCA patients have significantly lower exercise capacity compared to control subjects, possibly due to symptomatic angina reducing activity levels and ultimately exercise capacity (31–33), and/or ARD-related comorbidities. Randomized control trials have demonstrated supervised exercise training can improve coronary microvascular function, as assessed by CFR on doppler echocardiography (34), as well as endothelial function, as measured by brachial flow-mediated dilation (35). Conversely, deconditioning and lack of exercise may exacerbate CMD. Moreover, the systemic effects of ARD, such as musculoskeletal or pulmonary involvement, may converge with cardiac disease symptoms to cause worse functional capacity among CMD patients with ARD. It is possible that reduced exercise capacity, as demonstrated by impaired functional capacity, may be a consequence.

### Study limitations

There are several limitations to our study. Although ARD diagnosis was confirmed, initial assessment of ARD history was cross-sectional through self-report in the WISE-CVD questionnaire and may not capture the entire subgroup of patients who ultimately developed ARD, and medical records were obtained at enrollment. In addition, disease severity or disease duration of ARD was not available from chart review. Since we focused only among women with INOCA and confirmed CMD, we were unable to measure prevalence of CMD in general populations of ARD. Our analysis is not generalizable to men, to all INOCA patients, or to those with obstructive CAD. In addition, most of the patients with ARD in our study had SLE, thus may limit the generalizability to patients with other ARD types. Lastly, the sample size of CMD patients with ARD was small (*n* = 19), thus limiting the statistical power of the study.

## Conclusions

Among women with CMD, women with a history of ARD had lower functional status and worse myocardial perfusion reserve compared to women without ARD. Angina-related health status and invasive coronary function were not significantly different between groups. Further studies are warranted to understand mechanisms contributing to CMD among a larger cohort of women and men with ARDs with INOCA, as well as the role of immunotherapy in modulating CMD in various ARD populations.

## Data Availability

The raw data supporting the conclusions of this article will be made available by the authors, without undue reservation.
